# Targeted high-throughput sequencing of candidate genes for chronic obstructive pulmonary disease

**DOI:** 10.1186/s12890-016-0309-y

**Published:** 2016-11-11

**Authors:** Hans Matsson, Cilla Söderhäll, Elisabet Einarsdottir, Maxime Lamontagne, Sanna Gudmundsson, Helena Backman, Anne Lindberg, Eva Rönmark, Juha Kere, Don Sin, Dirkje S. Postma, Yohan Bossé, Bo Lundbäck, Joakim Klar

**Affiliations:** 1 Department of Biosciences and Nutrition, Karolinska Institutet, 7-9, SE-141 83 Huddinge, Sweden; 2 Department of Women’s and Children’s Health, Karolinska Institutet, Stockholm, Sweden; 3 Molecular Neurology Research Program, University of Helsinki and Folkhälsan Institute of Genetics, Helsinki, Finland; 4 Institut universitaire de cardiologie et de pneumologie de Québec, Québec, Canada; 5 Department of Immunology, Genetics and Pathology, Science for Life Laboratory, Uppsala University, Uppsala, Sweden; 6 Department of Public Health and Clinical Medicine, Division of Occupational and Environmental Medicine, Umeå University, Umeå, Sweden; 7 Department of Public Health and Clinical Medicine, Division of Medicine, Umeå University, Umeå, Sweden; 8 The University of British Columbia Center for Heart Lung Innovation, St-Paul’s Hospital, Vancouver, Canada; 9 Center Groningen, GRIAC research institute, University of Groningen, Groningen, The Netherlands; 10 Department of Molecular Medicine, Laval University, Québec, Canada; 11 Krefting Research Centre, Institute of Medicine, University of Gothenburg, Gothenburg, Sweden

**Keywords:** COPD, Sequencing, eQTL, Association, Lung development, *CHRNA5*

## Abstract

**Background:**

Reduced lung function in patients with chronic obstructive pulmonary disease (COPD) is likely due to both environmental and genetic factors. We report here a targeted high-throughput DNA sequencing approach to identify new and previously known genetic variants in a set of candidate genes for COPD.

**Methods:**

Exons in 22 genes implicated in lung development as well as 61 genes and 10 genomic regions previously associated with COPD were sequenced using individual DNA samples from 68 cases with moderate or severe COPD and 66 controls matched for age, gender and smoking. Cases and controls were selected from the Obstructive Lung Disease in Northern Sweden (OLIN) studies.

**Results:**

In total, 37 genetic variants showed association with COPD (*p* < 0.05, uncorrected). Several variants previously discovered to be associated with COPD from genetic genome-wide analysis studies were replicated using our sample. Two high-risk variants were followed-up for functional characterization in a large eQTL mapping study of 1,111 human lung specimens. The C allele of a synonymous variant, rs8040868, predicting a p.(S45=) in the gene for cholinergic receptor nicotinic alpha 3 (*CHRNA3*) was associated with COPD (*p* = 8.8 x 10^−3^). This association remained (*p* = 0.003 and OR = 1.4, 95 % CI 1.1-1.7) when analysing all available cases and controls in OLIN (*n* = 1,534). The rs8040868 variant is in linkage disequilibrium with rs16969968 previously associated with COPD and altered expression of the *CHRNA5* gene. A follow-up analysis for detection of expression quantitative trait loci revealed that rs8040868-C was found to be significantly associated with a decreased expression of the nearby gene cholinergic receptor, nicotinic, alpha 5 (*CHRNA5*) in lung tissue.

**Conclusion:**

Our data replicate previous result suggesting *CHRNA5* as a candidate gene for COPD and rs8040868 as a risk variant for the development of COPD in the Swedish population.

**Electronic supplementary material:**

The online version of this article (doi:10.1186/s12890-016-0309-y) contains supplementary material, which is available to authorized users.

## Background

Chronic obstructive pulmonary disease (COPD), characterised by a persistent airflow obstruction [1], is a life-threatening disease accounting for 6 % of all deaths globally in 2012 [2]. The development of the disease is influenced by environmental determinants, most commonly cigarette smoking, genetic risk factors and possible genetic protective factors [3]. Candidate gene association studies have suggested several potential COPD susceptibility genes, and genome-wide association studies (GWAS) have identified multiple COPD susceptibility loci [4]. However, genetic mapping in families with high penetrance for a disease gene variant can be helpful in pinpointing new susceptibility genes even for multifactorial traits. Recently, we reported mutations in the gene for fibroblast growth factor 10 (*FGF10*) involved in lung development, as a possible cause of COPD in families from Sweden [5]. Hence, a monogenic form of COPD could result from mutations in *FGF10*. To date, the only other known monogenic form of COPD is alpha 1-antitrypsin deficiency caused by disruption of the alpha-1-antiproteinase (*SERPINA1*) gene [6].

Typically in GWAS, common polymorphisms are tested for association. In this study, we provide an alternative approach with the aim to perform an in-depth analysis of exons of candidate genes for COPD by using high-throughput sequencing. This allowed us to detect the full spectrum of single nucleotide variation at any frequency in selected genomic regions and to also capture variants with a potential functional effect on gene expression levels. We show here that targeted high throughput sequencing using a well-defined population-based case–control sample can i) assess the impact of common variants in genes important for lung development, and ii) test genetic variants in a large set of candidate genes and genomic regions for association with COPD. To accomplish this we captured and sequenced 22 genes implicated in lung development as well as 61 genes and 10 genomic regions previously associated with COPD. The sample used here is comprised of cases and controls from The Obstructive Lung Disease in Northern Sweden (OLIN) studies. The population in northern Sweden, an admixture of three different ethnic groups (Swedes, Finns and Saami), showed a dramatic growth of population size since the 18^th^ century from a relatively small founder population [7]. This resulted in founder effects that significantly reduced the heterogeneity of this population, making it suitable for genetic association studies of multifactorial phenotypes, such as COPD [8].

This study assessed Swedish COPD cases and controls and assessed detected variants in candidate genes for association with COPD. We replicated a previous described association signal in CHRNA3, which also associated with lower *CHRNA5* gene expression. The DNA capture design and targeted sequencing used here show potential to detect known single nucleotide variants in association with COPD with the additional potential to also detect low-frequent variants. The result presented here using the relative limited sample size could be replicated using our targeted capture design in larger samples from different populations.

## Methods

### Patient material and ethics statement

The OLIN studies are an on-going research program focused on asthma, allergy and COPD. It started 30 years ago [9] and now involves more than 50,000 subjects from northern Sweden. Within OLIN, a COPD-cohort was identified at re-examination of several cohorts in 2002–2004 [10]. At recruitment, COPD (*n* = 993) was defined using the fixed ratio of FEV_1_ / FVC < 0.70 (forced expiratory volume in 1 s / forced vital capacity). When calculating the ratio FEV_1_ / FVC, the highest values of FEV_1_ and the highest value of forced vital capacity (FVC) or slow vital capacity (SVC) were used. This has support in the GOLD documents [1] and is acknowledged in the recent ERS task force guidelines for epidemiological studies on COPD [11]. An age and gender matched control population (*n* = 993) without obstructive lung function impairment was also recruited [10]. Since 2005 the OLIN COPD cohort with corresponding controls is followed up annually with a basic program including spirometry and interviews regarding symptoms and morbidity [12]. We initially selected 96 COPD cases (18 non-smokers, 43 former smokers and 35 smokers) from those who had an FEV_1_ < 80 % of predicted value in 2005 and either FEV_1_ / FVC < LLN (lower limit of normal) in 2010 or were rapid decliners with an annual FEV_1_ decline of ≥ 60 ml between 2005 and 2010. We also identified a set of 96 age- and gender-matched controls (33 smokers and 63 former smokers) with normal lung function. These 96 cases and 96 controls are henceforth termed the OLIN discovery sample (Table 1). Furthermore, we defined an OLIN replication sample consisting of individuals from the OLIN COPD study for which DNA was available (*n* = 1,534). From this group we classified individuals as cases when FEV_1_ / FVC was lower than LLN in 2010, or if they had a yearly FEV_1_ decline from 2005 to 2010 of at least 60 ml (*n* = 256). The remaining individuals were used as a reference group (*n* = 1,278). The physiological parameters for the OLIN replication sample included average age (cases 64 years, SD 11; controls 66 years, SD 11; *P* = 0.0012), gender (cases 123 females, 133 males; controls 569 females, 709 males; *P* = 0.30), smoking habits (cases 11 pack/year, SD 15; controls 23 pack/year, SD 17; *P* = 4.2 × 10^−9^), weight (cases 73 kg, SD 15; controls 77 kg, SD 14; *P* = 1.2 × 10^−4^) and height (cases 168 cm, SD 9; controls 168 cm, SD 10; *P* = 0.8). The phenotype description of this sample included measures of FVC (cases 3.29, SD 1.01; controls 3.50, SD 1. 03; *P* = 0.0031), FEV_1_ (cases 1.90, SD 0.67; controls 2.64, SD 0.81; *P* = 9.5 × 10^−43^) and FEV_1_% of predicted values (cases 67 %, SD 17 %; controls 95 % SD 16 %; *P* = 5.3 × 10^−73^), as well as the FEV_1_/FVC ratio (cases 0.57, SD 0.08; controls 0.75, SD 0.07; *P* = 2.0 × 10^−108^) when investigated the fifth year of the evaluations. For description of individual reference values, see Additional file 1.

**Table 1:** Pulmonary function in patients and controls

Average	FVC (cm^3^)	FVC LLN (cm^3^)	FVC pred (cm^3^)	FEV1 (cm^3^)	FEV1 LLN (cm^3^)	FEV1 pred (cm^3^)	FEV1%pred (%)	FEV1 / FVC	FEV1 / FVC LLN	FEV / FVC pred
Cases	2.69 (0.95)	2.87 (0.81)	3.79 (0.81)	1.50 (0.59)	2.01 (0.55)	2.78 (0.61)	53 (14)	0.56 (0.11)	0.62 (0.03)	0.73 (0.03)
Controls	3.96 (0.90)	2.77 (0.77)	3.83 (0.77)	3.11 (0.69)	2.05 (0.55)	2.82 (0.59)	110 (9)	0.79 (0.05)	0.62 (0.03)	0.74 (0.03)
*P* value	6.3 × 10^−18^	0.42	0.75	4.0 × 10^−41^	0.62	0.58	2.5 × 10^−81^	2.5 × 10^−45^	0.24	0.23

Values in parenthesis denote standard deviations. *FVC* Forced vital capacity, *LLN* Lower limit of normal, *FVC* pred, predicted FVC based on age, gender and length in the population. *FEV1* Forced expiratory volume in 1 s, *FEV1* Predicted FEV1 based on age, gender and length in the population, FEV1%pred, *FEV1* Divided by predicted FEV1 in the population, FEV1 / FVC ratio when investigated the fifth year of the evaluations. The lower limit of normal (LLN) values where calculated by subtracting 1.645 × RSD (residual standard deviation) from the predicted value (pred). FEV1%pred is the measured FEV1 value divided by the predicted value (i.e., FEV1 / FEV1pred). *P* values are calculated using two-sided Student’s *t*-test assuming equal variance. *SD* Standard deviation

*P* values for differences in parameters between cases and controls were calculated using two-sided Student’s t-tests, assuming equal variance. The ethics board of Umeå University (Dnr 04-045 M, supplement approved 2005-06-13) approved the use of individual phenotypic data and DNA samples for genetic research.

### Sequencing and quality controls

**Fig. 1:**
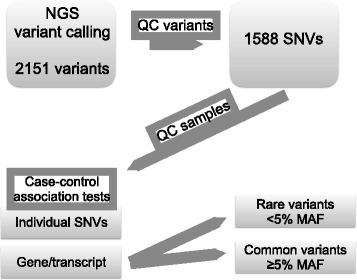
Flow chart of the strategy for variant calling, quality control and association tests. QC = quality control

In total 22 genes implicated in lung development, 61 genes and 10 genomic regions previously associated with COPD (Additional files 1 and 2), were investigated using targeted sequencing of captured genomic regions (HaloPlex Protocol Version A, Agilent Technologies, Santa Clara, CA). Regions of 1.5 kb of genomic sequence, including specific intergenic polymorphisms, was also included in the design. The regions of interest (ROI) were designed to target all known exons of major/known transcripts and at least 20 base pairs (bp) of intronic sequences flanking each exon. The sequence capture design included 953 target regions spanning 204.384 bp with 95.9 % (196.066 bp) coverage an average. Captured genomic regions were subjected to high throughput paired-end (100 bp read module) sequencing (HiSeq2000, Illumina, San Diego, CA) at the Science for Life Laboratory in Uppsala, Sweden. Sequence reads were aligned to the hg19 reference genome and single nucleotide variants (SNVs) were called using GATK Unified Genotyper (GATK bundle v.2.2) [13]. Next, we enriched for high quality SNVs by removing SNVs with low confidence (QD < 1.5), Phred scaled quality score (<50) and SNVs within SNV clusters. These high quality variants are henceforth referred to as ‘variants’. We also removed individual cases and controls with sequencing read depth consistently < 10 reads. The strategy for filtering and quality controls are illustrated in Fig. 1.

### Statistical analyses

Test for genetic association and genetic effect was performed for each predicted variant separately using the discovery sample. In addition, rs8040868 and rs11728716 were tested for association using the available OLIN sample (replication sample) as, according to RegulomeDB, these two markers present with potential functional effects on gene regulation (Table 2). Tests for allelic association of individual variants with COPD were performed using the Fisher’s exact test. Results were considered statistically significant when *p* < 0.05. No adjustment for multiple testing was performed in these analyses. Effect size was measured using odds-ratios (OR) with 95 % confidence intervals (CI).

**Table 2:** Associated genetic variants in the discovery sample set (*n* = 96 cases and *n* = 96 controls)

Chr	Pos (bp)	SNP rs ID	Alt	Ref	F c (%)	F p (%)	OR (95 % CI)	*P* value	Gene	Coding	RG score	ExAc Eur frequency^a^	CADD_PHRED^b^
1	110230569	72989301	G	A	10.0	22.1	2.5 (1.0-6.2)	0.038	*GSTM1*		4	0.24	NA
1	110233057	111436983	C	T	11.6	26.7	2.8 (1.2-6.2)	0.013	*GSTM1*		7	0.24	NA
2	216991935	12694384	A	C	29.4	17.9	0.5 (0.3-0.9)	0.04	*XRCC5*		5	NA	NA
2	218669225	61741262	C	T	13.6	0	NA	0.027	*TNS1*	p.(Asp1722Ser)	4	0.13	12.49
2	218746990	2303381	T	A	7.7	1.5	0.2 (0.0-0.9)	0.019	*TNS1*		6	NA	NA
3	55520778	566926	T	G	39.2	25.0	0.5 (0.3-0.9)	0.017	*WNT5A*		3a	NA	NA
4	106638697	72671840	G	A	2.3	11.4	5.3 (1.5-18.9)	5.8 × 10^−3^	*GSTCD*		6	NA	NA
4	106647679	72671858	T	C	3.0	10.5	3.7 (1.2-11.7)	0.026	*GSTCD*		7	NA	NA
4	106755996	11728716	A	G	3.4	21.0	7.4 (2.5-22.5)	6.5 × 10^−5^	*GSTCD*		1f	NA	NA
5	58284208	3805557	C	T	22.0	11.9	0.5 (0.2-0.9)	0.034	*PDE4D*		7	NA	NA
5	58284283	3805556	G	A	22.0	11.8	0.5 (0.2-0.9)	0.033	*PDE4D*		6	0.84	NA
5	58286625	1553114	C	T	22.0	11.8	0.5 (0.2-0.9)	0.033	*PDE4D*		7	0.84	NA
5	141993867	17223611	T	C	10.6	3.0	0.3 (0.1-0.8)	0.025	*FGF1*		5	NA	NA
6	142703137	2143390	T	C	2.9	19.2	7.9 (1.6-37.6)	4.5 × 10^−3^	*GPR126*	p.(Asp373=)	7	0.12	NA
6	151197501	9322290	C	T	17.4	9.0	0.5 (0.2-1.0)	0.047	*MTHFD1L*		5	NA	NA
6	151206894	147872265	T	C	7.6	2.2	0.3 (0.1-1.0)	0.049	*MTHFD1L*		7	0.002	NA
6	151263456	803451	A	G	41.8	55.7	1.8 (1.1-2.9)	0.04	*MTHFD1L*		7	NA	NA
6	151264132	803448	T	C	37.9	50.7	1.7 (1.0-2.7)	0.037	*MTHFD1L*		6	NA	NA
6	152183551	1643821	A	G	25.8	39.0	1.8 (1.1-3.1)	0.026	*ESR1*		6	NA	NA
8	42552530	41272375	C	G	1.5	6.9	4.8 (1.0-22.8)	0.034	*CHRNB3*		5	NA	NA
9	98239503	3780573	A	G	18.3	8.7	0.4 (0.2-0.9)	0.041	*PTCH1*		5	NA	NA
10	81706281	6413520	G	A	0.8	5.9	8.2 (1.0-66.4)	0.036	*SFTPD*	p.(Ser45=)	5	0.07	NA
10	123358096	41301039	G	C	25.0	2.8	0.1 (0.0-0.7)	0.017	*FGFR2*		4	NA	NA
11	102738499	632009	T	C	29.6	50.8	2.5 (1.5-4.1)	6.7 × 10^−4^	*MMP12*		7	NA	NA
12	23737566	11046992	A	G	21.2	33.1	1.8 (1.1-3.2)	0.039	*SOX5*		6	NA	NA
12	110224916	60258652	T	C	2.4	10.5	4.7 (1.0-22.9)	0.05	*TRPV4*		5	NA	NA
12	110224922	1861810	A	C	36.6	53.8	2.0 (1.1-3.8)	0.04	*TRPV4*		5	NA	NA
12	110232032	59870578	A	G	7.8	1.6	0.2 (0.0-0.9)	0.034	*TRPV4*		4	NA	NA
12	110232034	59940634	T	G	7.8	1.6	0.2 (0.0-0.9)	0.034	*TRPV4*		4	NA	NA
15	71434029	2004101	A	T	0.8	5.6	7.5 (0.9-61.6)	0.035	*THSD4*		7	NA	NA
15	78790189	2292115	G	A	4.8	0	NA	0.03	*IREB2*		7	NA	NA
15	78911181	8040868	C	T	31.8	47.8	2.0 (1.2-3.2)	8.8 × 10^−3^	*CHRNA3*	p.(Val53=)	1f	0.41	NA
16	16130514	903880	A	C	15.9	27.2	2.0 (1.1-3.6)	0.027	*ABCC1*		4	NA	NA
16	16205741	9673292	C	G	4.5	0	NA	0.013	*ABCC1*		6	NA	NA
16	16230290	212087	A	G	38.6	52.2	1.7 (1.1-2.8)	0.028	*ABCC1*		5	0.44	NA
16	16235366	113328089	A	G	6.9	1.5	0.2 (0.0-1.0)	0.034	*ABCC1*		5	NA	NA
20	15967390	41275442	T	C	9.1	17.7	2.1 (1.0-4.5)	0.049	*MACROD2*	p.(Thr100Met)	4	0.18	3.98

Alt, non-reference allele. Ref, reference allele, *OR* Odds ratio with 95 % confidence intervals, *F c* Frequency in controls, *F p* Frequency in cases (patients), *RG* Score, RegulomeDB score, *NA* Not available

^a^Non-Finnish European allele frequency extracted from ExAc v.0.3.1

^b^The “PHRED-scaled” CADD score based on ranks of all SNV in the hg19 genome reference

Visualization SNPs associated with COPD located in the CHRNA3/5 region was made using LocusZoom v1.1 [14] available on http://locuszoom.sph.umich.edu/locuszoom/. RefSeq gene/transcript case–control tests for aggregation of genetic variants in the targeted genomic regions were performed using PLINK/SEQ v0.1 [15]. Tests were divided into an analysis of rare variants with minor allele frequency (MAF) < 5 % or common variants (MAF ≥ 5 %). The UNIQ test, which identifies unique risk alleles, was utilized using default parameters to count the total number of alleles found only in cases (risk variants). Similarly, the SKAT burden tests, which assesses excess of rare alleles in cases compared to controls, was also utilized. Since both UNIQ and SKAT burden are 1-sided tests, we also swapped the phenotype information and analyzed the effects in both directions (excess of alleles in cases or controls) separately to capture evidence of both risk and protective alleles. Due to the matched design of the case and control groups no covariate adjustments (age, sex, pack-years) were performed in analysis using the discovery sample.

Linkage disequilibrium information of associated variants was extracted from genotypes from the sequencing analysis using Haploview 4.2 [16].

### Functional analysis of associated variants

To study the possible effect of associated variants on gene expression, we used information from RegulomeDB, a database that combines ENCODE data sets (chromatin immunoprecipitation sequencing (ChIP-seq) peaks, DNase I hypersensitivity peaks, DNase I footprints) with additional data sources (ChIP-seq data from the NCBI Sequence Read Archive, conserved motifs, expression quantitative trait loci (eQTL), and experimentally validated functional variants) [17]. A scoring system is based on the confidence of the functionality of variants, a lower score corresponding to stronger confidence. Subcategories are used to denote additional functional annotations. Combined Annotation Dependent Depletion (CADD) scores were used to assess potential structural and functional effect of associated nonsynonymous variants [18].

### Lung expression quantitative trait loci analyses

The existence of expression quantitative trait loci (eQTLs) was investigated as previously described using genotyping and gene expression data from 1,111 patients who underwent lung surgery at one of three sites, Laval University (discovery sample), University of British Columbia, and University of Groningen (replication sample sets) (referred to as Laval, UBC, and Groningen) [19, 20]. The eQTL data is derived from non-tumour lung parenchymal samples and expression data were adjusted for age, gender, and smoking status. Estimated *P*-values for each region were Bonferroni-corrected for multiple testing based on the number of SNPs and probe sets (number of SNPs x number of probe sets) and were considered significant if corrected *p* < 0.05.

### SNP genotyping for validation of rs11728716 and rs8040868

Individuals from the OLIN replication sample (*n* = 1,534) were genotyped for the rs11728716 and rs8040868 variants (99.2 % and 99.8 % success rate, respectively) at the Uppsala Genome Center (Uppsala, Sweden) using commercially available TaqMan assays (Life Technologies, Carlsbad, CA). Assay conditions were according to manufacturer’s recommendations. Effect size was estimated by comparing ORs with 95 % CI between cases and controls. Furthermore, to assess smoking dependence, we measured association and effect sizes also between the groups ‘non-smokers’ and ‘ever smokers’, and between ‘current smokers’ and ‘former smokers’.

## Results

### Selection of cases and controls for the discovery sample

The characteristics of each sample are listed in this section as value ± standard deviation. Cases and controls were matched for age (cases: 68 ± 10 years; controls: 66 ± 11 years; *p* = 0.15), gender (cases: 35 females, 61 males; controls: 31 females, 65 males; *p* = 0.65) and smoking habits (cases: 26 ± 19 pack/year; controls: 28 ± 12 pack/year; *p* = 0.53). Both groups were also closely matched for weight (cases: 76 ± 15 kg; controls: 77 ± 15 kg; *p* = 0.85) and height (cases: 169 ± 9 cm; controls: 169 ± 9 cm; *p* = 0.7). No non-smokers were included in the control group to avoid false negative results. The cases presented a significant reduction in lung function consistent with moderate or severe COPD. This is illustrated by a reduced FVC (cases: 2.69 ± 0.95 L; controls: 3.96 ± 0.90 L; *p* = 6.3 × 10^−18^), FEV_1_ (cases: 1.50 ± 0.59 L; controls: 3.11 ± 0.69 L; *p* = 4.0 × 10^−41^) and FEV_1_% of predicted values (cases: 53 ± 14 %; controls: 110 ± 9 %; *p* = 2.5 × 10^−81^), as well as the FEV_1_/FVC ratio (cases: 0.56 ± 0.11; controls: 0.79 ± 0.05; *p* = 2.5 × 10^−45^) when investigated the fifth year of the evaluations (Table 1).

### Test for association between genetic variants and COPD

We identified 2,151 SNVs after analysis of the sequenced target regions. After variant and sample quality control procedures, 1588 SNVs and 68 cases and 66 controls were retained in the downstream analysis (Fig. 1). Out of the 1588 variants, we identified 37 variants with significantly different allele frequencies in cases and controls (henceforth referred to as ‘associated variants’) (Table 2). We initially detected two novel variants in the discovery sample: GRCh37.p13, 5:g.157002804C > G in the *ADAM19* gene and GRCh37.p13, 7:g.73477874C > A in *ELN*. However, using Sanger sequencing of the same sample we excluded both variants, as they were monomorphic.

Three of the associated variants were shown to be unique to controls including missense variant rs61741262 (p.Asn1722Ser) in *TNS1*. The most significantly associated variants were all intronic (*GSTCD*, rs11728716, *p* = 6.5 × 10^−5^, OR = 7.4 (2.5-22.5) and *MMP12*, rs632009, *p* = 6.7 × 10^−4^, OR = 2.5 (1.5-4.1).

Although the majority of the associated variants were intronic (or intergenic), five were protein-coding (Table 2). Of these, two variants predicted amino-acid substitutions (missense variants): p.(Thr100Met) in *MACROD2* and p.(Asn1722Ser) in *TNS1* respectively. The p.(Asn1722Ser) variant could be potential damaging based on a relative high CADD score or 12.49. Of the coding variants, we found that rs2143390, predicting a p.(D373=) in *GPR126* (*p* = 0.005, OR = 7.9 (1.6-37.6)), rs6413520 in *SFTPD* (p.(Ser45=), *p* = 0.036, OR = 8.2 (1.0-66.4)), rs8040868 in *CHRNA3* (p.(Val53=), *p* = 8.8 × 10^−3^, OR = 2.0 (1.2-3.2)) and rs41275442 in *MACROD2* (p.(Thr100Met), *p* = 0.049, OR = 2.1 (1.0-4.5)) conferred moderate to high risk for COPD (Table 2).

We tested the presence of variants uniquely found in cases or controls as well as gene burden tests in cases against controls as specified in PLINK/SEQ v0.1 using standard settings. We noted that the *ADAM19*, *WNT2*, *CHRNA5*, *NOS3* and *PTCH1* genes all harbor rare variants (MAF < 5 %) uniquely found in cases (Additional file 3). Conversely, the *FGF8*, *CTNNB1* and *HHIP* genes contain rare variants uniquely found in the control sample (Additional file 3). Neither gene burden analysis (SKAT) or analysis of rare alleles (MAF < 5 %) yielded significant results. However, by performing a joint analysis with only common alleles (MAF ≥ 5 %) in target regions using SKAT, we showed a significant gene burden for the genes *GSTCD*, *FGF1*, *ELN* and *ESR1* (Additional file 4).

### Haplotypes and linkage disequilibrium

We identified five regions with associated variants in pairwise LD (r^2^ > 0.7, D´ = 1.0). The regions were located at the *GSTM1* gene locus on chromosome 1 (rs72989301-rs111436983), *GSTCD* on chromosome 4 (rs72671840-rs72671858), *PDE4D* on chromosome 5 (rs3805557- rs3805556- rs1553114), *MTHFDIL* on chromosome 6 (rs803451- rs803448) and *TRPV4* on chromosome 12 (rs59870578-rs59940634) (Additional file 5). Furthermore, the variant rs8040868 on chromosome 15q21.1 is in pairwise LD (r^2^ = 0.76, D’ = 1.0) with rs16969968, a nonsynonymous variant previously associated with expression of the *CHRNA5* gene [21]. The rs16969968 variant was included in our capture design but it did not reach significant association in the OLIN discovery sample (OR 1.6; *p* = 0.07) (Additional file 6).

### *In silico* analysis of predicted functions of associated variants

According to RegulomeDB, all 37 associated variants were located within known and predicted regulatory elements in intergenic regions (Table 2). We noted that a variant in *CHRNA3* (rs8040868) and a variant in *GSTCD* (rs11728716) each showed a RegulomeDB score of “1f”, denoting the presence of transcription binding site or DNAse peak.

### Lung eQTL results

**Fig. 2:**
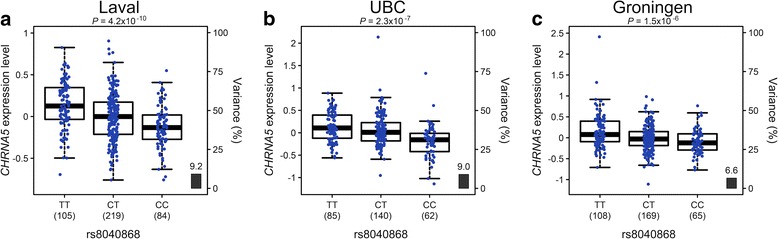
*CHRNA5* gene expression levels in the lungs according to genotype groups for rs8040868. The left y-axis represents standardised gene expression levels in the lung with heterozygous genotype group set to zero. The x-axis represents the three genotyping groups, TT, CT and CC (risk allele C), for the variant in (**a**) the discovery set (Laval) *n* = 408; *P* = 4.2 × 10^−10^, and the replication sets (**b**) UBC, *n* = 287; *P* = 2.31 × 10^−7^, and (**c**) Groningen, *n* = 342; *P* = 1.5 × 10^−6^. The number of subjects per genotype group is indicated in parenthesis. The right y-axis shows the proportion of the gene expression variance explained by the variant (black bar)

According to RegulomeDB, the rs8040868 (*CHRNA3)* and rs11728716 (*GSTCD)* variants could present with potential functional effects on gene regulation. To determine if these variant could represent eQTL, we analysed the genotypes and gene expression data in the discovery sample (Laval) as well as replication samples (UBC and Groningen). One of these variants, rs8040868:C > T, was confirmed to be significantly associated with gene expression of the nearby gene *CHRNA5* in all three data sets, with the C allele (minor allele) associated with lower *CHRNA5* expression (Fig. 2 and Additional file 7). Interestingly, we could also see a high correlation between rs8040868 and expression of an anti-sense transcript (AF147302) of unknown function from the adjacent *IREB2* gene region (data not shown). AF147302 is likely a result of strong bi-directional promoter activity in this region [22].

### The rs8040868 variant is associated with COPD in the OLIN replication sample

We also investigated pulmonary data from the replication sample (*n* = 1,534; cases = 256, controls = 1278). Analysis using RegulomeDB predicted both rs11728716 and rs8040868 variants as being functional (score 1f for both variants). We therefore selected these two variants for genotyping in all available OLIN samples (*n* = 1,534). The frequency of the rs8040868-C allele was 35 % in the reference group (*n* = 1,278) and 42 % in the cases (*n* = 256) resulting in a significant association (*p* = 0.003) and an OR of 1.4 (95 % CI 1.1-1.7) for COPD. SweGen variant frequency database reports a 39 % frequency of rs8040868 in 1000 whole genomes representing a cross-section of the Swedish population (https://swefreq.nbis.se). The frequency of the homozygous rs8040868-CC genotype was 12 % in the reference group and significantly higher (18 %) in the cases (*p* = 0.018). When comparing smoking status using all genotyped individuals, no significant difference in allele frequency between neither the groups ‘non-smokers’ (*n* = 589) and ‘ever smokers’ (*n* = 943) (OR 1.1 95 % CI 1.0-1.7; *p* = 0.09) nor between the groups ‘current smokers’ (*n* = 312) and ‘former smokers’ (*n* = 631) (OR 1.2 95 % CI 1.0-1.4; *p* = 0.11) was seen. The latter test was used to assess nicotine dependency and aptitude for smoking cessation under the assumption that a genetic variant associated with these traits would be underrepresented in a former smoking group as compared to a group of current smokers, i.e., harder to quit smoking. The tests for association with smoking must however be taken with caution as the confidence intervals are wide and a larger sample size would be needed for replication.

Analysis of rs11728716 using the OLIN replication sample (*n* = 1,534) revealed no association with COPD (*p* = 0.07; OR = 2.2). In order to test if rs11728716 is associated with severe COPD, we stratified the available COPD cases based on severity and selected cases with FEV1%pred < 40 % and FEV1/FVC < LLN. Our results show a significant association (*p* = 0.017) between rs11728716 and the group of severe COPD (*n* = 14). The allele frequency of rs11728716-A was 10 % among cases with severe COPD and 4 % in the controls.

## Discussion and conclusion

Genetic variants influencing lung function in children and adults may ultimately lead to the development of COPD [23]. Since limited disease-specific therapy for COPD is available, an improved knowledge of genetic variants modulating the pathogenic mechanisms underlying COPD is greatly needed. We aimed here to identify genetic variants within, or close to, the coding regions of genes and loci previously associated with COPD, or in genes involved in lung development. We opted for a qualitative rather than a quantitative approach with the selection of cases with moderate or severe COPD and progressive decline in lung function. Furthermore, controls were all smokers without COPD that, in our study design, can aid the identification of potential protective genetic variants and aid detection of genetic variants associated with severe COPD. When applying a Bonferroni correction for the total number of variants detected, no variants showed statistically significant association. We did, however, identify several variants with a likely biological significance, as indicated by high effect sizes (odds ratio), that we believe warrants further investigation in a larger sample. Furthermore, potential functional effects of variants were investigated using data from a large number of lung samples and we describe here a COPD lung eQTL.

When comparing our association data with the lung eQTL data (discovery data set from Laval University), we could identify a variant associated with COPD that was also associated with level of gene expression (Fig. 2). This variant, synonymous variant (rs8040868) in *CHRNA3* on chromosome 15, confers a risk for the development of COPD in both our OLIN discovery sample with moderate or severe COPD and our OLIN replication sample including all available COPD cases and controls in OLIN (OR 1.4, *p* = 0.003). In the lung eQTL data, we could see a correlation of the C allele of rs8040868 with lower expression levels of *CHRNA5* (Fig. 2), and, to a lesser extent, also *CHRNA3* and *PSMA4*, which are located in close proximity to *CHRNA5*. The α-nicotinic receptor (*CHRNA3/5*) gene locus on chromosome 15q25.1 is associated with COPD, lung cancer and peripheral arterial disease, as well as other smoking related conditions [24, 25] and nicotine addiction [26, 27]. Recently, the *CHRNA3/5* locus was implicated in all-cause mortality among smokers in a Finnish cohort [2]. The rs8040868-C allele associates with both reduced pulmonary function and lung cancer [24, 25, 28, 29] and affects DNA-methylation and transcription of *CHRNA5* [30]. Furthermore, rs8040868 is also in LD with a nearby variant (rs16969968) previously reported to be associated with expression levels of *CHRNA5* in the lung [21]. The direction of effect is the same for both SNPs, with the minor alleles associated with reduced expression of *CHRNA5*. Also recently, rs16969968 was found to be the most significantly associated variant in an exome array analysis in a study including more than 6,100 COPD cases and 6,000 control subjects across five cohorts [31].

Several genetic variants showed association with COPD in our population, but did not correlate with gene expression levels in the lung, including previously identified variants in the genes glutathione S-transferase, c-terminal domain containing (*GSTCD*), surfactant protein D (*SFTPD*) and matrix metalloproteinase-12 (*MMP12*) [32–36]. We identified a haplotype consisting of three risk-conferring variants, rs72671840, rs72671858 and rs11728716 (G-T-A haplotype), at the *GSTCD* gene locus on chromosome 4q24. The variant rs11728716 has previously been associated with lung function [32–34] and is likely to affect the transcription of *GSTCD.* We show here that rs11728716 was associated with severe COPD using the OLIN replication sample. Although intriguing, due to the limited number of severe COPD cases used in this study, this result needs further verification in a larger sample. The other two variants (rs72671840 and rs72671858) are of unknown function [37]. *GSTCD* encodes a glutathione S-transferase C-terminal domain protein involved in detoxification by catalysing conjugation of glutathione to products of oxidative stress. We found association between COPD and rs6413520, a synonymous variant, p.(Ser45=), within *SFTPD* on chromosome 10q22.3*.* This variant conferred a high risk (OR = 8.2) for COPD in our study and has previously been reported to be associated with COPD susceptibility [36]. *SFTPD* encodes surfactant protein D, of importance for the regulation of oxidant production, inflammatory responses, and apoptotic cell clearance in the lung [38]. We also identified rs632009, in the *MMP12* gene on chromosome 11q22.3, to confer moderate risk. Matrix metalloproteinases (MMPs) are involved in both tissue remodelling and repair and several members of the MMP family have been implicated in COPD pathology [35, 39, 40].

In this study, we also found association (uncorrected) with novel susceptibility variants. Several variants in the G-protein-coupled receptor 126 (*GPR126*) gene on chromosome 6q24.1 have previously been associated with FEV_1_ / FVC ratio [32]. GPR126 belongs to a superfamily of G protein-coupled receptors and is involved in cell signalling and adhesion. Studies in mice show an induction of Gpr126 expression between embryonic day 7 and 11 with expression in the developing heart and face as well as a high expression in the adult lung [41]. We found significant association between a synonymous variant in the *GPR126* gene (rs2143390, p.(Asp373=)) and COPD. The alternative T allele is highly overrepresented in cases compared to controls (*p* = 4.5 × 10^−3^, OR = 7.9).

We also focused our attention to the chromosome 4q31 locus upstream of *HHIP*, previously shown to be associated with expression of the gene [20, 42]. The *HHIP* upstream region belongs to one of the so far strongest COPD association signals [43], but no association could be seen in our case–control groups for any upstream variants.

The sequencing approach allowed us to detect rare alleles in both cases and controls. We therefore performed gene burden tests to find evidence of overrepresented rare or common variants in individual genes or transcripts in the cases or controls, respectively. Interestingly, we found that the genes *ADAM19*, *WNT2*, *CHRNA5*, *NOS3* and *PTCH1* all contain rare variants (MAF < 5 %) uniquely found in cases of the OLIN discovery sample. These variants, and especially the coding variants with predicted functional effect, could be followed up in a larger case–control sample for verification and further genetic and functional analysis.

We assessed 83 genes and 10 genomic regions of 1.5 kb size for variants associated with COPD in a sample from Northern Sweden. Still, one limitation of our study is that the targeted capture design may exclude yet unknown genomic regions that can harbour genetic variation influencing COPD. Also, the two novel variants detected after sequencing were monomorphic and an assessment of the false discovery rate using HaloPlex with subsequent Illumina sequencing would be helpful in order to evaluate our set of candidate genes as a gene panel for COPD. Furthermore, we cannot rule out that some findings are influenced by population substructure and replication of our result in different populations is essential. It is also possible that some risk variants were not identified due to the limited number of cases and controls used for sequencing. Using a conservative Bonferroni correction based on the 1588 variants detected resulted in no variants reached significant association with COPD. However, we believe there is no definite consensus regarding the type of multiple testing procedures to use in targeted sequencing based approaches. Furthermore, many parameters such as variant quality checks, genotyping success rate and sequencing depth limit will influence the number of variants found, and consequently, multiple testing adjustments. Also, in addition to include genes including variants previously associated with COPD or asthma, we explored if a set of genes involved in lung development would harbour variants in association with COPD in the Swedish discovery sample. Therefore, as the study is exploratory with a mixed hypothesis the *p* values for association testing in this study are not corrected for multiple testing.

Despite the limited size of the discovery sample used here, we identified several high-risk genetic variants for COPD and we replicated several previous GWAS results. In particular, our results support the *CHRNA5* gene as a likely candidate gene for COPD where the rs8040868-C allele confers a risk for the disease in the Swedish population. Furthermore, we indicate the advantage of using less heterogeneous populations in the studies of complex disorders.

## Additional files

Additional file 1: Supplementary methods. Detailed methods description regarding classification of COPD, sequencing protocol and linkage disequilibrium analysis. (DOCX 123 kb)
Additional file 2: Targeted genes and variants for targeted high throughput sequencing. The table lists the selected genes and variants included in the study. (DOCX 79 kb)
Additional file 3: Number of rare variants found uniquely in cases and controls. The table present data of the UNIQ test of rare variants in cases versus controls. (DOCX 78 kb)
Additional file 4: Gene burden analysis of common variants. The table present the SKAT analysis of gene burden of common variants. (DOCX 86 kb)
Additional file 5: Pairwise linkage disequilibrium (LD) of associated variants. A list of detected genetic variants found to be in LD. (DOCX 46 kb)
Additional file 6 A plot containing genomic positions and *p*-values of variants in the *CHRNA3/CHRNA5* gene locus with rs8040869 and rs16969968 highlighted. (PDF 144 kb)
Additional file 7 Probe sets replicated in both replication sets (UBC and Groningen) in the lung eQTL analyses. A table of replicated probe sets in the lung eQTL analysis. (DOCX 38 kb)

## Abbreviations

ChIP-seq: Chromatin immunoprecipitation sequencing
COPD: Chronic Obstructive Pulmonary Disease
eQTLs: Expression quantitative trait loci
FEV_1_: Expiratory volume in 1 s FVC: forced vital capacity
GWAS: Genome-wide association studies
LLN: Lower limit of normal
OLIN: The Obstructive Lung Disease in Northern Sweden studies
SNVs: Single nucleotide variants forced
SVC: Slow vital capacity
