# Supplementary material for: Targeted high-throughput sequencing of candidate genes for chronic obstructive pulmonary disease
Source: BMC Pulm Med. 2016 Nov 11;16:146. doi: 10.1186/s12890-016-0309-y (PMC5106844; doi:10.1186/s12890-016-0309-y)
Supplement: Additional file 1: — Supplementary methods. Detailed methods description regarding classification of COPD, sequencing protocol and linkage disequilibrium analysis. (DOCX 123 kb) [file 12890_2016_309_MOESM1_ESM.docx]

Supplementary methods

**Reference values for lung function and classification of COPD**

Individual reference values (predicted values) for lung function were calculated according to Roberts et al [[1](#_ENREF_1)]. The reference values are comparable to previously published values from the Swedish population [[2-4](#_ENREF_2)]. The definition of COPD was based on the lower limit of normal (LLN) values for FEV_1_/FVC as recommended by the American Thoracic Society (ATS) and the European Respiratory Society (E) (ATS/E 2005) [[5](#_ENREF_5)]. This is based on the normal distribution and classifies the bottom 5% of the healthy population as deviant. The LLN range was calculated by subtracting 1.645 x D (residual standard deviation) from the predicted value.

**Sequencing and quality controls**

The genomic regions of interest were designed to target all exons and transcript variants including small intragenic introns of the genes (1-83) and 1.5 kb genomic sequence centered on specific intergenic variants (84-93) (Additional file 2). HaloPlex library preparations were performed according to protocol. First, 900 ng of high-molecular weight genomic DNA per sample was digested with 16 different restriction endonucleases. Control of sufficient digestion was performed using agarose gel electrophoresis. Capture probes were hybridized to digested DNA and amplified followed by product clean-up. Size estimation of individual libraries was performed using a Bioanalyzer 2100 instrument with high sensitivity DNA assays (Agilent Technologies, Santa Clara, CA). Novel single nucleotide variants associated with COPD were verified by Sanger sequencing in all 96 cases and 96 controls used as the discovery sample set. Primers were designed using Primer3plus [[6](#_ENREF_6)]. Regions of interest for capture and primer sequences are available upon request.

**Haplotypes and linkage disequilibrium**

Pairwise linkage disequilibrium (LD) analysis was performed using SNAP and the variant data set from the CEU population panel of the 1000 genome project [[7](#_ENREF_7)]. The r^2^ threshold was set to 0.7 and the distance limit to 500 kb. Haplotypes for each individual were confirmed by manual investigation of genotypes of adjacent variants in LD. We further tested LD between rs8040868 and variants previously reported to affect expression of the *CHRNA5* gene at the same locus (rs16969968. rs12907966 and rs8034191) [[8](#_ENREF_8)].

**References**

1 C. M. Roberts, K. D. MacRae, A. J. Winning, L. Adams and W. A. Seed. Reference values and prediction equations for normal lung function in a non-smoking white urban population. Thorax. 1991;46:643-50.

2 E. Berglund, G. Birath, J. Bjure, G. Grimby, I. Kjellmer, L. Sandqvist, et al. Spirometric studies in normal subjects. I. Forced expirograms in subjects between 7 and 70 years of age. Acta Med Scand. 1963;173:185-92.

3 H. Hedenstrom, P. Malmberg and K. Agarwal. Reference values for lung function tests in females. Regression equations with smoking variables. Bull Eur Physiopathol Respir. 1985;21:551-7.

4 H. Hedenstrom, P. Malmberg and H. V. Fridriksson. Reference values for lung function tests in men: regression equations with smoking variables. Ups J Med Sci. 1986;91:299-310.

5 R. Pellegrino, G. Viegi, V. Brusasco, R. O. Crapo, F. Burgos, R. Casaburi, et al. Interpretative strategies for lung function tests. Eur Respir J. 2005;26:948-68.

6 A. Untergasser, H. Nijveen, X. Rao, T. Bisseling, R. Geurts and J. A. Leunissen. Primer3Plus, an enhanced web interface to Primer3. Nucleic Acids Res. 2007;35:W71-4.

7 A. D. Johnson, R. E. Handsaker, S. L. Pulit, M. M. Nizzari, C. J. O'Donnell and P. I. de Bakker. SNAP: a web-based tool for identification and annotation of proxy SNPs using HapMap. Bioinformatics. 2008;24:2938-9.

8 J. D. Nguyen, M. Lamontagne, C. Couture, M. Conti, P. D. Pare, D. D. Sin, et al. Susceptibility loci for lung cancer are associated with mRNA levels of nearby genes in the lung. Carcinogenesis. 2014;35:2653-9.
