# Supplementary material for: Targeted high-throughput sequencing of candidate genes for chronic obstructive pulmonary disease
Source: BMC Pulm Med. 2016 Nov 11;16:146. doi: 10.1186/s12890-016-0309-y (PMC5106844; doi:10.1186/s12890-016-0309-y)
Supplement: Additional file 2: — Targeted genes and variants for targeted high throughput sequencing. The table lists the selected genes and variants included in the study. (DOCX 79 kb) [file 12890_2016_309_MOESM2_ESM.docx]

**Targeted genes and variants for targeted high throughput sequencing.**

| # | Gene name | Implication in COPD | References |
| --- | --- | --- | --- |
| 1 | *FGF10* | Lung developmental gene | Klar et al 2011. Shi et al 2009 |
| 2 | *FGF7* | Lung developmental gene | Shi et al 2009. Sauleda et al 2008 |
| 3 | *FGF8* | Lung developmental gene | Shi et al 2009 |
| 4 | *FGF9* | Lung developmental gene | Shi et al 2009 |
| 5 | *FGF1* | Lung developmental gene | Kranenburg et al 2005. Shi et al 2009 |
| 6 | *FGF2* | Lung developmental gene | Kranenburg et al 2005. Shi et al 2009 |
| 7 | *FGFR1* | Lung developmental gene | Kranenburg et al 2005. Shi et al 2009 |
| 8 | *FGFR2* | Lung developmental gene | Shi et al 2009 |
| 9 | *VEGFA* | Lung developmental gene | Pavlisa et al 2010. Siafakas et al 2007 |
| 10 | *SHH* | Lung developmental gene | Shi et al 2009 |
| 11 | *HHIP* | Lung developmental gene | Hancock et al 2010. Pillai et al 2009. Artigas et al 2011 |
| 12 | *TGFB1* | Lung developmental gene | Chappell et al 2011. Castaldi et al 2010. Shi et al 2009. van Diemen et al 2010 |
| 13 | *CEBPA* | Lung developmental gene | Didon et al 2010 |
| 14 | *CEBPB* | Lung developmental gene | Roos et al 2012 |
| 15 | *BMP4* | Lung developmental gene | Ninomiya et al 2013 |
| 16 | *DKK1* | Lung developmental gene | Shu et al 2005 |
| 17 | *ALDH1A2* | Lung developmental gene | Shi et al 2009 |
| 18 | *STAT3* | Lung developmental gene | Qu et al 2009 |
| 19 | *HGF* | Lung developmental gene | Sauleda et al 2008 |
| 20 | *WNT2* | Lung developmental gene | Wang et al 2011 |
| 21 | *WNT5a* | Lung developmental gene | Wang et al 2011 |
| 22 | *WNT7b* | Lung developmental gene | Wang et al 2011 |
| 23 | *ABCC1* | COPD associated gene | Obeidat et al 2011 |
| 24 | *ACE* | COPD associated gene | Hopkinson et al 2004 |
| 25 | *ADAM19* | COPD associated gene | Hancock et al 2010 |
| 26 | *ADAM33* | COPD associated gene | Simpson et al 2005. Gosman et al 2007 |
| 27 | *ADRB2* | COPD associated gene | Hegab et al 2004 |
| 28 | *AGER* | COPD associated gene | Artigas et al 2011 |
| 29 | *BICD1* | COPD associated gene | Kong et al 2011 |
| 30 | *CHRNA3* | COPD associated gene | Pillai et al 2009 |
| 31 | *CHRNA5* | COPD associated gene | Pillai et al 2009 |
| 32 | *CHRNB3* | COPD associated gene | Cho et al 2010 |
| 33 | *CHRNB4* | COPD associated gene | Cho et al 2010 |
| 34 | *CNTN5* | COPD associated gene | Obeidat et al 2011 |
| 35 | *PTGS2* | COPD associated gene | Arif et al 2008 |
| 36 | *CTNNB1* | COPD associated gene | Garcia Lunan et al 2009 |
| 37 | *DEFB1* | COPD associated gene | Andresen et al 2011 |
| 38 | *ELN* | COPD associated gene | Wan et al 2010 |
| 39 | *EPHX1* | COPD associated gene | Lee et al 2011. Lakhdar et al 2011. Putra et al 2013 |
| 40 | *ESR1* | COPD associated gene | Obeidat et al 2011 |
| 41 | *FAM13A* | COPD associated gene | Hancock et al 2010. Cho et al 2010 |
| 42 | *GPR126* | COPD associated gene | Hancock et al 2010 |
| 43 | *GSTCD* | COPD associated gene | Repapi et al 2010. Artigas et al 2011 |
| 44 | *GSTM1* | COPD associated gene | Castaldi et al 2010. Lakhdar et al 2011 |
| 45 | *GSTP1* | COPD associated gene | Vibhuti et al 2007. Zhong et al 2011. Lakhdar et al 2011 |
| 46 | *GSTT1* | COPD associated gene | Lakhdar et al 2011 |
| 47 | *HMOX1* | COPD associated gene | Putra et al 2013 |
| 48 | *HTR4* | COPD associated gene | Hancock et al 2010. Repapi et al 2010. Artigas et al 2011 |
| 49 | *IL13* | COPD associated gene | Hegab et al 2004 |
| 50 | *IL1B* | COPD associated gene | Danilko et al 2007. Lee et al 2008 |
| 51 | *IL1RN* | COPD associated gene | Danilko et al 2007 |
| 52 | *IL4* | COPD associated gene | Hegab et al 2004 |
| 53 | *IL6* | COPD associated gene | Danilko et al 2007 |
| 54 | *IL10* | COPD associated gene | Demeo et al 2008 |
| 55 | *IREB2* | COPD associated gene | DeMeo et al 2009. Chappell et al 2011 |
| 56 | *LTA4H* | COPD associated gene | Danilko et al 2007 |
| 57 | *MACROD2* | COPD associated gene | Obeidat et al 2011 |
| 58 | *MMP1* | COPD associated gene | Korytina et al 2008. Zhang et al 2005 |
| 59 | *MMP12* | COPD associated gene | Korytina et al 2008. Hunninghake et al 2009. Zhang et al 2005 |
| 60 | *MMP9* | COPD associated gene | Korytina et al 2008. Zhang et al 2005 |
| 61 | *MTHFD1L* | COPD associated gene | Obeidat et al 2011 |
| 62 | *NOS1* | COPD associated gene | Dupont et al 2014 |
| 63 | *NOS2* | COPD associated gene | Dupont et al 2014 |
| 64 | *NOS3* | COPD associated gene | Dupont et al 2014 |
| 65 | *PPT2* | COPD associated gene | Hancock et al 2010 |
| 66 | *PDE4D* | COPD associated gene | Obeidat et al 2011 |
| 67 | *PID1* | COPD associated gene | Hancock et al 2010 |
| 68 | *PTCH1* | COPD associated gene | Hancock et al 2010 |
| 69 | *SERPINA1* | COPD associated gene | Obeidat et al 2011. Chappell et al 2006 |
| 70 | *SERPINA3* | COPD associated gene | Demeo et al 2008 |
| 71 | *SERPINE2* | COPD associated gene | Fujimoto 2010 |
| 72 | *SFTPA1* | COPD associated gene | van Diemen et al 2010 |
| 73 | *SFTPA2* | COPD associated gene | van Diemen et al 2010 |
| 74 | *SFTPB* | COPD associated gene | van Diemen et al 2010 |
| 75 | *SFTPD* | COPD associated gene | van Diemen et al 2010 |
| 76 | *SOD3* | COPD associated gene | Castaldi et al 2010 |
| 77 | *SOX5* | COPD associated gene | Heh et al 2011 |
| 78 | *THSD4* | COPD associated gene | Repapi et al 2010. Artigas et al 2011 |
| 79 | *TIMP2* | COPD associated gene | Ghanei et al 2010 |
| 80 | *TNF* | COPD associated gene | Castaldi et al 2010. Danilko et al 2007. Demeo et al 2008 |
| 81 | *TNS1* | COPD associated gene | Repapi et al 2010. Artigas et al 2011 |
| 82 | *TRPV4* | COPD associated gene | Obeidat et al 2011 |
| 83 | *XRCC5* | COPD associated gene | Heh et al 2010 |
| 84 | *TGFB1* | COPD associated variant. 1800470 | Castaldi et al 2010 |
| 85 | *TGFB1* | COPD associated variant. 1800469 | van Diemen et al 2010 |
| 86 | *TNF* | COPD associated variant. 1800629 | Castaldi et al 2010 |
| 87 | *PTGS2* | COPD associated variant. 20417 | Arif et al 2008 |
| 88 | *HHIP* | COPD associated variant. 12504628 | Repapi et al 2010 |
| 89 | *MACROD2* | COPD associated variant. 204652 | Obeidat et al 2011 |
| 90 | *CNTN5* | COPD associated variant. 17133553 | Obeidat et al 2011 |
| 91 | *MTHFD1L* | COPD associated variant. 803450 | Obeidat et al 2011 |
| 92 | *ABCC1* | COPD associated variant. 3887893 | Obeidat et al 2011 |
| 93 | *ESR1* | COPD associated variants. 11155818/9478248 | Obeidat et al 2011 |
