# Supplementary material for: Targeted high-throughput sequencing of candidate genes for chronic obstructive pulmonary disease
Source: BMC Pulm Med. 2016 Nov 11;16:146. doi: 10.1186/s12890-016-0309-y (PMC5106844; doi:10.1186/s12890-016-0309-y)
Supplement: Additional file 3: — Number of rare variants found uniquely in cases and controls. The table present data of the UNIQ test of rare variants in cases versus controls. (DOCX 78 kb) [file 12890_2016_309_MOESM3_ESM.docx]

**Number of rare variants found uniquely in cases and controls.**

| Transcript | Pos | Gene | NVar | Test | *P* value | Cases/controls |
| --- | --- | --- | --- | --- | --- | --- |
| Case unique variants <5% MAF | |  |  |  |  |  |
| NM_033274 | chr5:156908148..157002695 | *ADAM19* | 21 | UNIQ | 0.004 | 18/0 |
| NM_003391 | chr7:116917956..116918235 | *WNT2* | 2 | UNIQ | 0.013 | 1/0 |
| NR_024047 | chr7:116917956..116918235 | *WNT2* | 2 | UNIQ | 0.013 | 1/0 |
| NM_000745 | chr15:78880577..78885369 | *CHRNA5* | 4 | UNIQ | 0.015 | 5/0 |
| NM_000603 | chr7:150690623..150709623 | *NOS3* | 18 | UNIQ | 0.022 | 18/0 |
| NM_001083604 | chr9:98209156..98268765 | *PTCH1* | 13 | UNIQ | 0.042 | 8/0 |
| NM_001083605 | chr9:98209156..98268765 | *PTCH1* | 13 | UNIQ | 0.042 | 8/0 |
| NM_001083606 | chr9:98209156..98268765 | *PTCH1* | 13 | UNIQ | 0.042 | 8/0 |
| NM_001083607 | chr9:98209156..98268765 | *PTCH1* | 13 | UNIQ | 0.042 | 8/0 |
| NM_000264 | chr9:98209156..98270362 | *PTCH1* | 14 | UNIQ | 0.045 | 8/0 |
| NM_001083602 | chr9:98209156..98279234 | *PTCH1* | 16 | UNIQ | 0.049 | 9/0 |
| NM_001083603 | chr9:98209156..98279234 | *PTCH1* | 16 | UNIQ | 0.049 | 9/0 |
| Control unique variants <5% MAF | |  |  |  |  |  |
| NM_001206389 | chr10:103529898..103535583 | *FGF8* | 9 | UNIQ | 0.009 | 8/0 |
| NM_006119 | chr10:103529898..103535583 | *FGF8* | 9 | UNIQ | 0.009 | 8/0 |
| NM_033163 | chr10:103529898..103535583 | *FGF8* | 9 | UNIQ | 0.009 | 8/0 |
| NM_033164 | chr10:103529898..103535583 | *FGF8* | 9 | UNIQ | 0.009 | 8/0 |
| NM_033165 | chr10:103529898..103535583 | *FGF8* | 9 | UNIQ | 0.009 | 8/0 |
| NM_001098209 | chr3:41266689..41280807 | *CTNNB1* | 5 | UNIQ | 0.011 | 5/0 |
| NM_001098210 | chr3:41266689..41280807 | *CTNNB1* | 5 | UNIQ | 0.011 | 5/0 |
| NM_001904 | chr3:41266689..41280807 | *CTNNB1* | 5 | UNIQ | 0.011 | 5/0 |
| NM_022475 | chr4:145567258..145656024 | *HHIP* | 11 | UNIQ | 0.014 | 10/0 |

Pos, genomic coordinates for the range of the corresponding locus. NVar, number of variants. Cases/controls, the number of cases/controls for with variants are detected.
