# Supplementary material for: Targeted high-throughput sequencing of candidate genes for chronic obstructive pulmonary disease
Source: BMC Pulm Med. 2016 Nov 11;16:146. doi: 10.1186/s12890-016-0309-y (PMC5106844; doi:10.1186/s12890-016-0309-y)
Supplement: Additional file 4: — Gene burden analysis of common variants. The table present the SKAT analysis of gene burden of common variants. (DOCX 86 kb) [file 12890_2016_309_MOESM4_ESM.docx]

**Gene burden analysis of common variants.**

| Transcript | Pos | Gene | Nvar | Test | *P* value | Cases/controls (number of variants) |
| --- | --- | --- | --- | --- | --- | --- |
| NM_001031720 | chr4:106638697..106755996 | *GSTCD* | 4 | SKAT | 0.009 | 14/4(1);15/3(1);21/4(1);5/10(1) |
| NM_024751 | chr4:106638697..106755996 | *GSTCD* | 4 | SKAT | 0.009 | 14/4(1);15/3(1);21/4(1);5/10(1) |
| NM_000800 | chr5:141974572..141993867 | *FGF1* | 3 | SKAT | 0.011 | 4/14(1);63/64(1);69/64(1) |
| NM_001144892 | chr5:141974572..141993867 | *FGF1* | 3 | SKAT | 0.011 | 4/14(1);63/64(1);69/64(1) |
| NM_001144934 | chr5:141974572..141993867 | *FGF1* | 3 | SKAT | 0.011 | 4/14(1);63/64(1);69/64(1) |
| NM_001144935 | chr5:141974572..141993867 | *FGF1* | 3 | SKAT | 0.011 | 4/14(1);63/64(1);69/64(1) |
| NM_001257205 | chr5:141974572..141993867 | *FGF1* | 3 | SKAT | 0.011 | 4/14(1);63/64(1);69/64(1) |
| NM_001257206 | chr5:141974572..141993867 | *FGF1* | 3 | SKAT | 0.011 | 4/14(1);63/64(1);69/64(1) |
| NM_001257207 | chr5:141974572..141993867 | *FGF1* | 3 | SKAT | 0.011 | 4/14(1);63/64(1);69/64(1) |
| NM_001257208 | chr5:141974572..141993867 | *FGF1* | 3 | SKAT | 0.011 | 4/14(1);63/64(1);69/64(1) |
| NM_001257209 | chr5:141974572..141993867 | *FGF1* | 3 | SKAT | 0.011 | 4/14(1);63/64(1);69/64(1) |
| NM_001257210 | chr5:141974572..141993867 | *FGF1* | 3 | SKAT | 0.011 | 4/14(1);63/64(1);69/64(1) |
| NM_001257211 | chr5:141974572..141993867 | *FGF1* | 3 | SKAT | 0.011 | 4/14(1);63/64(1);69/64(1) |
| NM_001257212 | chr5:141974572..141993867 | *FGF1* | 3 | SKAT | 0.011 | 4/14(1);63/64(1);69/64(1) |
| NM_033136 | chr5:141974572..141993867 | *FGF1* | 3 | SKAT | 0.011 | 4/14(1);63/64(1);69/64(1) |
| NM_000501 | chr7:73452140..73480805 | *ELN* | 12 | SKAT | 0.023 | 11/6(1);13/9(1);15/2(1);15/20(1);17/19(1);53/52(1);54/49(1);57/56(1);8/12(1);8/9(1);9/12(2) |
| NM_001081752 | chr7:73452140..73480805 | *ELN* | 12 | SKAT | 0.023 | 11/6(1);13/9(1);15/2(1);15/20(1);17/19(1);53/52(1);54/49(1);57/56(1);8/12(1);8/9(1);9/12(2) |
| NM_001081753 | chr7:73452140..73480805 | *ELN* | 12 | SKAT | 0.023 | 11/6(1);13/9(1);15/2(1);15/20(1);17/19(1);53/52(1);54/49(1);57/56(1);8/12(1);8/9(1);9/12(2) |
| NM_001081754 | chr7:73452140..73480805 | *ELN* | 12 | SKAT | 0.023 | 11/6(1);13/9(1);15/2(1);15/20(1);17/19(1);53/52(1);54/49(1);57/56(1);8/12(1);8/9(1);9/12(2) |
| NM_001081755 | chr7:73452140..73480805 | *ELN* | 12 | SKAT | 0.023 | 11/6(1);13/9(1);15/2(1);15/20(1);17/19(1);53/52(1);54/49(1);57/56(1);8/12(1);8/9(1);9/12(2) |
| NM_000125 | chr6:152129077..152420095 | *ESR1* | 10 | SKAT | 0.040 | 16/15(1);18/26(1);20/25(1);24/28(1);29/37(1);32/29(1);4/11(1);49/35(1);5/13(1);53/34(1) |
| NM_001122740 | chr6:152129077..152420095 | *ESR1* | 10 | SKAT | 0.040 | 16/15(1);18/26(1);20/25(1);24/28(1);29/37(1);32/29(1);4/11(1);49/35(1);5/13(1);53/34(1) |
| NM_001122741 | chr6:152129077..152420095 | *ESR1* | 10 | SKAT | 0.040 | 16/15(1);18/26(1);20/25(1);24/28(1);29/37(1);32/29(1);4/11(1);49/35(1);5/13(1);53/34(1) |
| NM_001122742 | chr6:152129077..152420095 | *ESR1* | 10 | SKAT | 0.040 | 16/15(1);18/26(1);20/25(1);24/28(1);29/37(1);32/29(1);4/11(1);49/35(1);5/13(1);53/34(1) |

Pos, genomic coordinates for the range of the corresponding locus. Nvar, number of variants.
