# Supplementary material for: Targeted high-throughput sequencing of candidate genes for chronic obstructive pulmonary disease
Source: BMC Pulm Med. 2016 Nov 11;16:146. doi: 10.1186/s12890-016-0309-y (PMC5106844; doi:10.1186/s12890-016-0309-y)
Supplement: Additional file 5: — Pairwise linkage disequilibrium (LD) of associated variants. A list of detected genetic variants found to be in LD. (DOCX 46 kb) [file 12890_2016_309_MOESM5_ESM.docx]

| SNP rs ID | Proxy SNP | Distance (bp) | r^2^ | D’ | Chromosome | Gene | Coordinate hg19 (bp) |
| --- | --- | --- | --- | --- | --- | --- | --- |
| rs72989301 | rs111436983 | 2488 | 0.87 | 1 | 1 | *GSTM1* | 110233057 |
| rs72671840 | rs72671858 | 8982 | 1 | 1 | 4 | *GSTCD* | 106638697 |
| rs3805557 | rs3805556 | 75 | 1 | 1 | 5 | *PDE4D* | 58284283 |
| rs3805557 | rs1553114 | 2417 | 1 | 1 | 5 | *PDE4D* | 58286625 |
| rs3805556 | rs1553114 | 2342 | 1 | 1 | 5 | *PDE4D* | 58286625 |
| rs803451 | rs803448 | 676 | 0.86 | 1 | 6 | *MTHFDIL* | 151264132 |
| rs59870578 | rs59940634 | 2 | 1 | 1 | 12 | *TRPV4* | 110232034 |

**Pairwise linkage disequilibrium (LD) of associated variants.**

SNP, query variant. Proxy SNP, proxy variant in LD with the query variant. Distance, genomic distance in bp between query and proxy variant. LD is described as

squared coefficient of correlation (r^2^) or deviation (D’).
