# Supplementary figures and images for: Targeted high-throughput sequencing of candidate genes for chronic obstructive pulmonary disease
Source: BMC Pulm Med. 2016 Nov 11;16:146. doi: 10.1186/s12890-016-0309-y (PMC5106844; doi:10.1186/s12890-016-0309-y)

Plotted SNPs

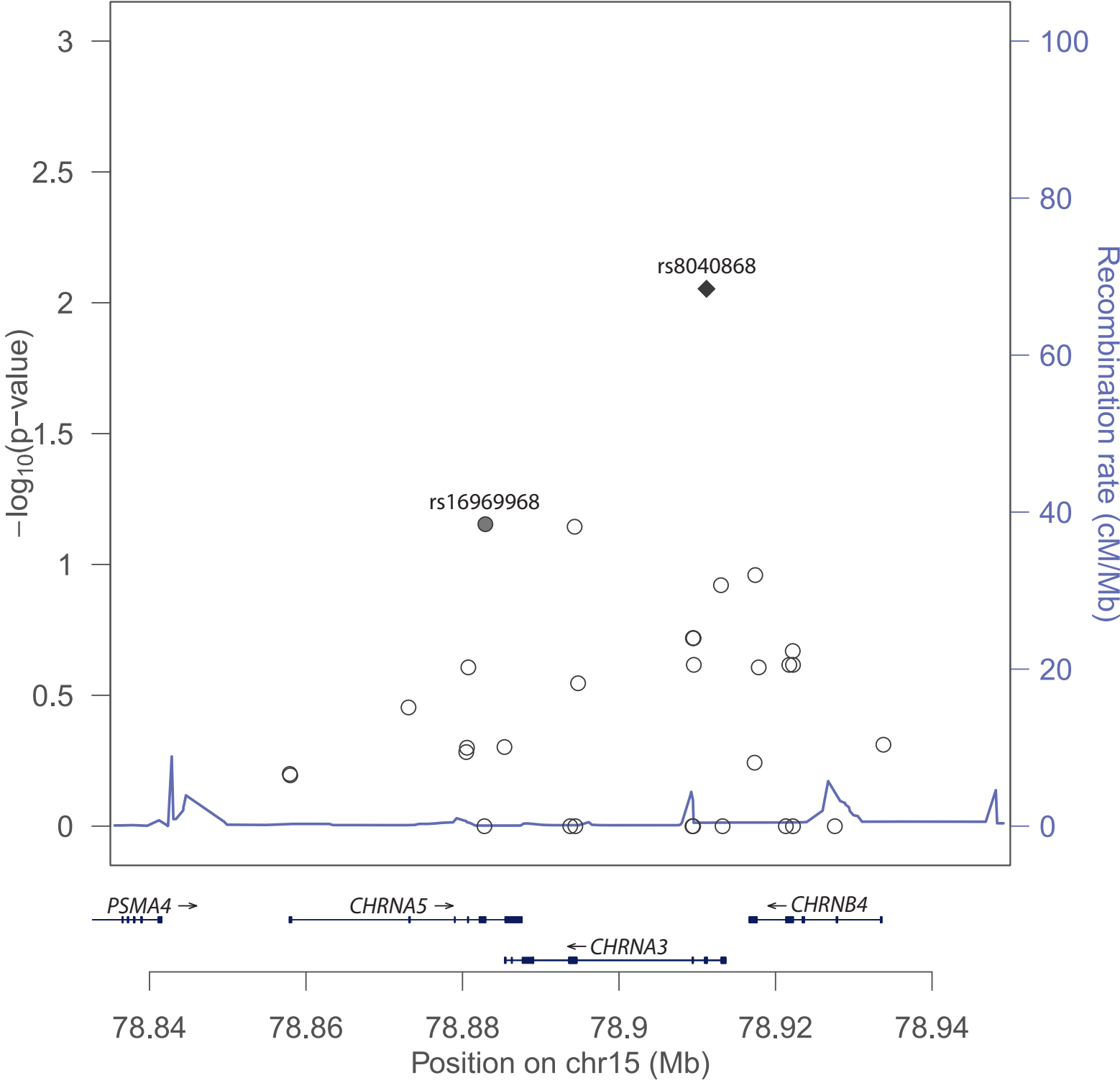

Supplement: Additional file 6 — A plot containing genomic positions and p-values of variants in the CHRNA3/CHRNA5 gene locus with rs8040869 and rs16969968 highlighted. (PDF 144 kb) [file 12890_2016_309_MOESM6_ESM.pdf]
