# Supplementary material for: Targeted high-throughput sequencing of candidate genes for chronic obstructive pulmonary disease
Source: BMC Pulm Med. 2016 Nov 11;16:146. doi: 10.1186/s12890-016-0309-y (PMC5106844; doi:10.1186/s12890-016-0309-y)
Supplement: Additional file 7 — Probe sets replicated in both replication sets (UBC and Groningen) in the lung eQTL analyses. A table of replicated probe sets in the lung eQTL analysis. (DOCX 38 kb) [file 12890_2016_309_MOESM7_ESM.docx]

**Probe sets replicated in both replication sets (UBC and Groningen) in the lung eQTL analyses.**

| ProbeSet | Variant | Chr. | Position | Meta *P-*value | Threshold | Gene | Accession | OR  (95% CI)* | *P* value* |
| --- | --- | --- | --- | --- | --- | --- | --- | --- | --- |
| 100154936_TGI_at | rs8040868 | 15 | 78911181 | 5.35x10^-39^ | 2.38x10^-04^ |  | AF147302 | 2.30  (1.42 -3.71) | 2.9x10^-4^ |
| 100156434_TGI_at | rs8040868 | 15 | 78911181 | 1.98x10^-19^ | 2.38x10^-04^ | *CHRNA5* | NM_000745 |  |  |

*, results from the association study. Thresholds indicate Bonferroni corrected significance level.
